# Seasonal Variation of Peritonsillar Abscess Presentation to an Emergency Department in an Atlantic Canadian Climate: A Retrospective Review

**DOI:** 10.1177/00034894221127485

**Published:** 2022-10-11

**Authors:** Kalpesh Hathi, Miranda X. Lees, Kavish Chandra, David Lewis, Ben McMullin, Christopher J. Chin

**Affiliations:** 1 Dalhousie Medicine New Brunswick, Saint John, New Brunswick, Canada; 2 Department of Emergency Medicine, Horizon Health Network, Saint John Regional Hospital, Saint John, New Brunswick, Canada; 3 Division of Otolaryngology-Head & Neck Surgery, Department of Surgery, Dalhousie University, Halifax, Nova Scotia, Canada

**Keywords:** peritonsillar abscess, infection, bacteria, epidemiology, seasonal variation

## Abstract

**Objectives:**

Patients with a peritonsillar abscess (PTA) often present to emergency departments as the first point of medical contact. Upper respiratory tract infections (URTIs) are more frequent in the winter. Therefore, we hypothesize that the incidence of PTAs will be more frequent in colder winter months as well. This is the first study assessing the seasonal variation and epidemiology of PTA presentations to an emergency department in Atlantic Canada, home to a unique maritime climate.

**Methods:**

A retrospective cohort study was conducted through a chart review of all patients who presented to the Saint John Regional Hospital Emergency Department from January 1, 2015, to December 31, 2020. Patient characteristics, treatment, and microbiology were reported. A chi-square goodness-of-fit test assessed the seasonal variation of PTA. Pearson correlations assessed PTA incidence per mean monthly temperature and humidity.

**Results:**

A total of 75 patients were included. 57.3% were male and 42.7% were female, with a mean age (±SD) of 35.9 ± 14.0. Most patients presented afebrile (82.7%, cutoff ≥ 38.0°C). Approximately half of all patients had an elevated WBC count (49.3%, cutoff ≥ 10.9 × 10^9^). The most common bacteria isolated were Streptococcus species followed by anaerobic bacteria (17.9%). No significant variation was found with respect to season (X^2^(3) = 1.0, *P* = .801), temperature (*r(70)* = 0.198, *P* = .096), or humidity, (*r(70)* = 0.063, *P* = .599).

**Conclusion:**

This study did not find a seasonal variation of PTA in a maritime climate. These findings question the anecdotal hypothesis that PTA is associated with progression from acute URTIs and therefore would be more common in the winter months.

## Background

Peritonsillar abscess (PTA) is an upper airway emergency in which pus collects between the superior constrictor muscle of the pharynx and the capsule of the palatine tonsil. Its pathogenesis has yet to be clearly defined. One suggestion is that PTA arises as a complication of an acute upper respiratory tract infection (URTI).^1^ Previous studies have shown PTA has an incidence of ~10 to 30/100,000, tends to occur in the third decade of life, and is the most common deep space neck infection.^1-4^ Both anaerobic and aerobic bacteria are commonly isolated from aspirated pus, with Streptococcus species and *Fusobacterium necrophorum* being commonly involved pathogens.^3^ The treatment of PTA involves abscess drainage, antimicrobials, and rehydration.^1-4^ Tonsillectomy is considered in patients with multiple episodes of PTA.^1-4^

The epidemiology of PTA has been rarely assessed in Canada, with the most recent papers published in 2016 and 1990.^2,5^ The epidemiology, microbiology, and antibiotic resistance patterns of PTA can differ over time and based on location.^6,7^ To the best of our knowledge, this has never been assessed in Atlantic Canada, the setting of this study.

It is hypothesized that the incidence of PTA would increase in colder/winter seasons and climates due to the increased incidence of acute URTIs.^1,8^ However, the literature remains mixed as to whether a seasonal variation of PTA incidence exists. Some studies report no significant seasonality.^2,9-13^ The studies that do show significant seasonality demonstrate varying tends, with some studies showing a higher incidence in warmer months and others showing a higher incidence in colder months.^14-16^ Regarding how seasonal variation is measured in the existing literature, few studies have assessed seasonality with respect to quantitative climate measures such as temperature and humidity. The studies that included these measures did not show a correlation between the incidence of PTA and humidity.^14^ Existing research has examined the seasonality of PTA in both tropical and temperate climates, but never in a maritime climate such as that in Atlantic Canada. Only one study has examined the seasonal variation of PTA in Canada; this study, conducted in London, Ontario, found no significant seasonal variability.^2^ No other studies to our knowledge have reported on this within North America.

Saint John, New Brunswick, is uniquely situated on the Bay of Fundy in Atlantic Canada. This location results in a milder maritime climate with noticeable seasonal variation, humid summers quickly change to colder winters. Given the climate of New Brunswick does not match that of the regions in which the seasonal variation of PTA has been studied thus far, in addition to the limited quantitative measuring of temperature and humidity with PTA incidence, it is challenging to apply the results of existing studies to a maritime climate and population. The literature shows mixed evidence of PTA seasonality, further highlighting the need for the seasonal variation of PTA incidence to be researched in a maritime climate. This study assesses the epidemiology and seasonal variation of PTA presentations at the Saint John Regional Hospital (SJRH) Emergency Department (ED), a tertiary care center in Atlantic Canada.

## Methods

Approval for retrospective data collection and a waiver of consent were obtained from the Horizon Health Network Research Ethics Board, ROMEO File #: 101227. Paper and electronic health records of all patients ≥19 years old with a discharge diagnosis of PTA from the SJRH ED from January 1, 2015 to December 31, 2020, were reviewed. Patients were excluded if they were found not to have a PTA when further assessed in the otolaryngology clinic. Only the first presentation was considered if patients presented to the SJRH ED multiple times with the same PTA. At least 1 month between presentations was required to be considered separate cases. Patients diagnosed with a PTA in the ED but reported to have an “early PTA,” a “resolving PTA,” or peritonsillar cellulitis when later assessed in the otolaryngology clinic were included in our cohort. PTAs were diagnosed clinically.

Patients <19 years old were excluded as they experience a wide range of unique risk factors for the development of URTIs, which are independent of season. Compared to adults, children spend more time in crowded environments including daycares, schools, and extracurricular activities which inherently increase their risk of contracting URTIs and by extent the potential development of PTAs, regardless of the season or climate. Further, a child’s immune system is still developing, making them more prone to viral and bacterial infections, independent of season or climate. As this study focuses on the impact of season, patients <19 years old were excluded to limit these potential confounding variables uniquely present in pediatric patients. Including pediatric patients would limit conclusions specific to seasonality.

The created database included the patients’ biological sex, age at the time of ED presentation, date and season of presentation, area of residence, smoking history, history of PTA, side of PTA, patient’s body temperature upon presentation to ED, white blood cell (WBC) count in the ED, PTA treatment, microbial culture results, antibiotic resistance, antibiotics prescribed, and whether the patient was registered with a primary care provider at the time of PTA presentation. Bacterial culture results were based on both throat swabs and culture of PTA aspiration. Antibiotics and steroids prescribed in the hospital were recorded, and the discharge antibiotic prescription was also recorded. Whether the patients resided in New Brunswick’s Health Zone 2, the zone primarily served by the SJRH ED, was also collected.^17^

The seasons were divided based on months, similar to our institution’s previous study.^18^ Summer was defined as June, July, and August; Fall was defined as September, October, and November; Winter was defined as December, January, and February; and Spring was defined as March, April, and May. Mean monthly temperature and humidity were collected from readings by Environment Canada at the Saint John Airport.^19^

### Data Analysis

All data were analyzed using SPSS Version 25.0. A priori power analyses were run for all proposed statistical analyses and confirmed our sample size is adequately powered to identify large effect sizes for the chi-square analysis (*w* = 0.61, minimum sample size 30) and moderate effect sizes for the Pearson correlation (*r* = .3, minimum sample size 67).^20^ A priori significance level of *P* < .05 was set for all statistical analyses.

Patient demographics, clinical presentation and treatment were reported descriptively. Categorical variables were described as absolute counts and percentages. Continuous variables were described as means ± standard deviation (SD).

The number of patients with an elevated WBC count (≥10.9 × 10^9^) was reported, and the number of patients who were febrile (≥38.0°C) was reported.

PTA cases at the SJRH ED were described as absolute counts and percentages per month and season. Seasonal variation of PTA was compared based on the number of cases across seasons using a chi-square goodness-of-fit test. The effect of humidity and temperature was assessed using Pearson correlations; the number of cases in a month was correlated to the mean monthly temperature and humidity. Only patients who resided within the New Brunswick Health Zone 2 were included in the temperature and humidity analysis to avoid variations based on the area of residence. All patients were included in the seasonal variation analysis as area of residence would not affect the season.

The number of cases per mean monthly temperature/humidity was analyzed to capture the average climate surrounding the development of the PTA. Average weekly or daily temperatures were not selected for analysis as it would increase our risk of autocorrelation and type one error.

## Results

### Patient Sample

**Figure 1.**
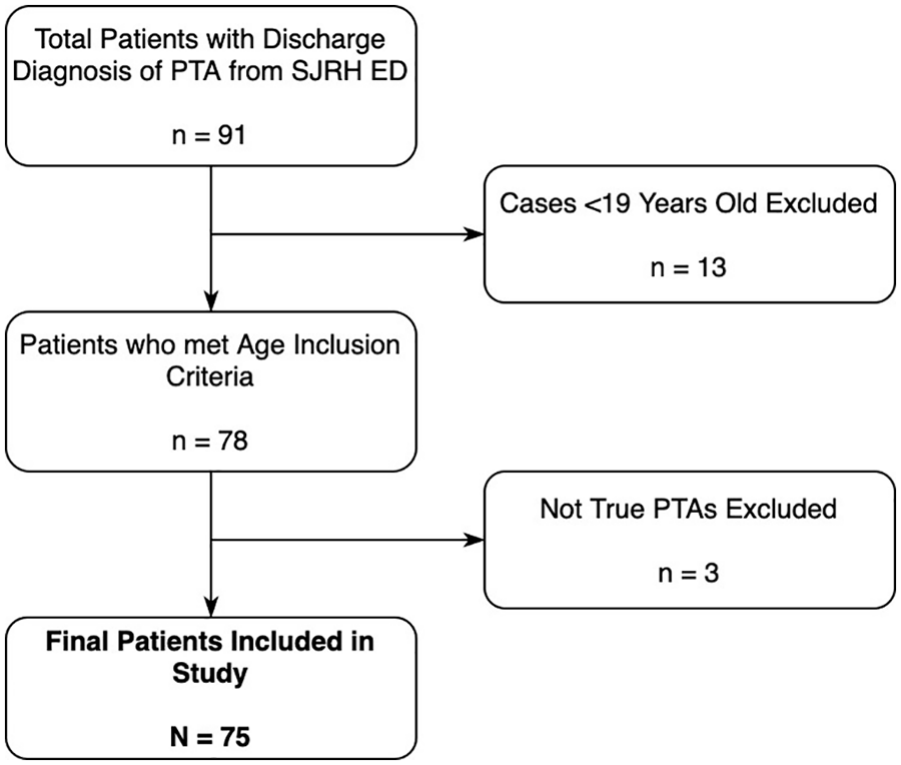
Flow diagram depicting patient inclusion and exclusion. Abbreviations: ED, emergency department; PTA, peritonsillar abscess.

A total of 91 patients had a discharge diagnosis of PTA from the SJRH ED between January 1, 2015, and December 31, 2020. Of these, 75 were included in this study; 13 were excluded for being <19 years old, and 3 were excluded for not being true PTAs (Figure 1). Of the 75 patients included in the final cohort, 68 patients residing within the New Brunswick Health Zone 2 were included in the Pearson correlation analysis for the effect of temperature and humidity. All 75 patients were included in all other analyses.

**Figure 2.**
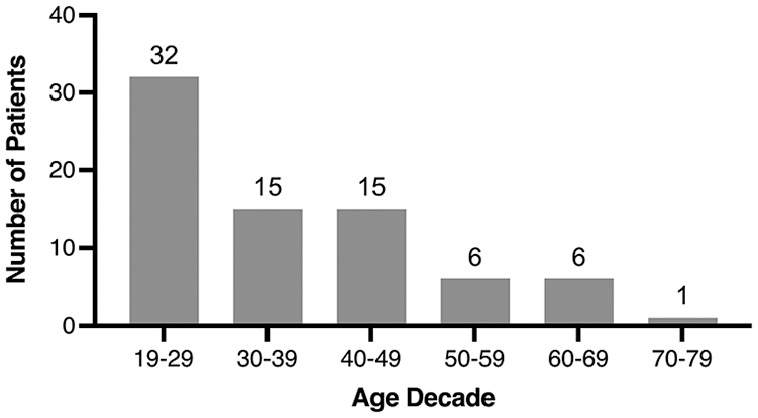
Age distribution of patients presenting with a peritonsillar abscess by decade.

Patient characteristics are summarized in Table 1. Of note, 49.3% of patients had an elevated WBC count. However, most patients were afebrile (82.7%) at ED triage. Most patients were registered with a primary care provider at presentation (82.7%). Our cohort had a slight male predominance (males 57.3%; females 42.7%). The side of the PTA presentation was almost equivalent (right 49.3%; left 48.0%). A history of prior PTA was reported in 9.3% of patients, and 16.0% of patients were reported to be current smokers. Figure 2 shows that most patients presenting with a PTA were in their third decade of life (19-29 years old; 42.7%).

**Table 1.** Descriptive Characteristics of Patients Presenting with a Peritonsillar Abscess.

Variable	N (%) n = 75
Age (Mean ± SD)	35.9 ± 14.0
Sex	
Male	43 (57.3)
Female	32 (42.7)
Side of PTA	
Right	37 (49.3)
Left	36 (48.0)
Unknown	2 (2.7)
Registered with a primary care provider	
Yes	62 (82.7)
No	13 (17.3)
History of PTA	
Yes	7 (9.3)
No/Not reported	68 (90.7)
Smoking	
Current smoker	12 (16.0)
Previous smoker	7 (9.3)
Non-smoker/Not reported	56 (74.7)
Patient temperature (Mean ± SD)	37.2°C ± 0.7°C
Febrile (>38.0°C)	10 (13.3)
Afebrile (<38.0°C)	62 (82.7)
Unknown	3 (4.0)
WBC (Mean ± SD)	14.1 × 10^9^ ± 4.5 × 10^9^
Elevated WBC (≥10.9 × 10^9^)	37 (49.3)
Normal WBC (<10.9 × 10^9^)	12 (16)
Unknown	26 (34.7)

Abbreviations: PTA, peritonsillar abscess; SD, standard deviation; WBC, white blood cell.

### Seasonal Variation

**Figure 3.**
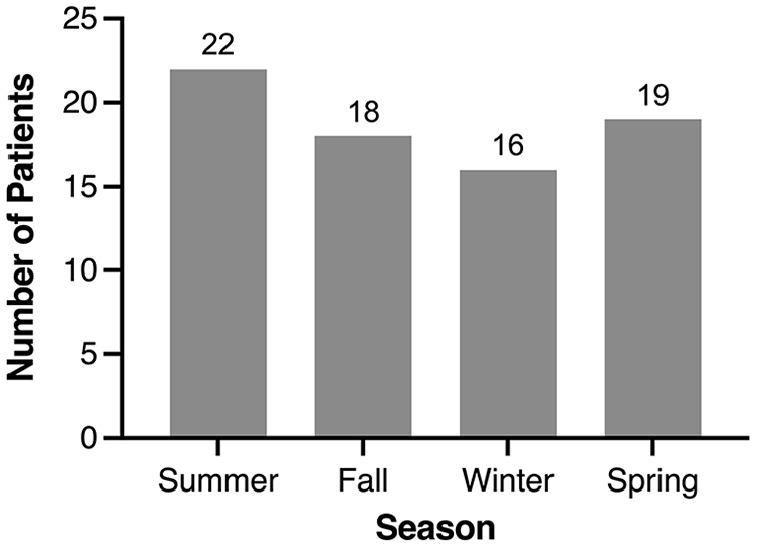
Seasonal distribution of patients presenting with a peritonsillar abscess.

Figure 3 and Table 2 show the number of cases of PTA at the SJRH ED per season and month. The greatest incidence of cases occurred during the summer (29.3%). However, the chi-square goodness-of-fit test did not show any significant differences in seasonal variation (X^2^(3) = 1.0, *P* = .801). The most common month of presentation was May (13.3%).

**Table 2.** Patients who Presented with a Peritonsillar Abscess by Months and Seasons.

Variable	N (%) n = 75
Season	
Summer	22 (29.3)
Fall	18 (24.0)
Winter	16 (21.3)
Spring	19 (25.3)
Month	
January	8 (10.7)
February	4 (5.3)
March	0 (0)
April	9 (12.0)
May	10 (13.3)
June	8 (10.7)
July	5 (6.7)
August	9 (12.0)
September	5 (6.7)
October	8 (10.7)
November	5 (6.7)
December	4 (5.3)

**Figure 4.**
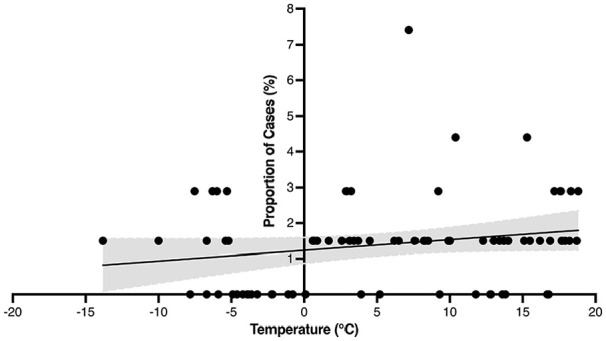
Proportion of peritonsillar abscess cases at mean monthly temperatures. Shading represents 95% CI.

**Figure 5.**
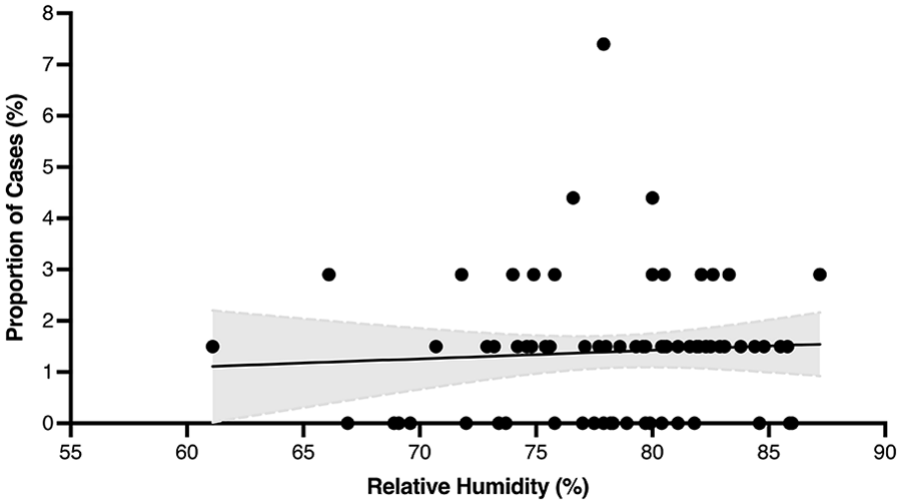
Proportion of peritonsillar abscess cases at mean monthly relative humidity. Shading represents 95% CI.

The proportion of PTA cases presenting in a month was plotted in relation to each month’s mean temperature and relative humidity. Figures 4 and 5 represent this graphically on scatter plots that show the proportion of PTA cases per mean monthly temperature and mean monthly relative humidity, respectively. The shaded areas represent the 95% confidence intervals (CI). Pearson correlational analyses showed no significant differences based on mean monthly temperature, *r(70)* = 0.198, *P* = .096 nor based on mean monthly relative humidity, *r(70)* = 0.063, *P* = .599. These graphics and analyses included only the cases within New Brunswick Health Zone 2 (n = 68).

### Treatment and Microbiology

The treatment of the patients in our series is summarized in Table 3. Most patients were given dexamethasone in the ED (65.3%). Clindamycin alone (54.7%) was the most common antibiotic given in the ED and the most common antibiotic prescribed on discharge (46.7%). 92% of patients were discharged from ED, and 8% were admitted.

**Table 3.** Treatment of Patients with a Peritonsillar Abscess.

Variable	N (%) n = 75
Length of hospital stay (Mean ± SD)	0.28 ± 1.1 day
Same day discharge	69 (92.0)
Admitted to hospital	6 (8.0)
Steroids prescribed in hospital	
Dexamethasone	49 (65.3)
Prednisone	1 (1.3)
Methylprednisolone	1 (1.3)
No steroid/Unknown	24 (32.1)
Antibiotics prescribed in hospital	
Clindamycin	41 (54.7)
Cefazoline & metronidazole	9 (12.0)
Amoxicillin/Clavulin	7 (9.3)
Clindamycin & amoxicillin	4 (5.3)
Ceftriaxone	4 (5.3)
Cefalexin	3 (4.0)
Azithromycin & ceftriaxone	1 (1.3)
Penicillin	1 (1.3)
No Antibiotic/Unknown	5 (6.7)
Discharge antibiotics	
Clindamycin	35 (46.7)
Amoxicillin/Clavulin	19 (25.3)
Cefalexin & metronidazole	9 (12.0)
Cefalexin	3 (4.0)
Azithromycin	3 (4.0)
Cefazolin	2 (2.7)
Clindamyzin & amoxicillin	1 (1.3)
Vancomycin & cefalexin	1 (1.3)
Unspecified	2 (2.7)

Abbreviations: SD, standard deviation.

A total of 39 (52.0%) patients within our cohort had a throat swab or bacterial culture completed. Table 4 summarizes the available antibiotic resistance trends and bacterial culture results from these 39 patients. *Streptococcus pyogenes* was the most common bacteria isolated (30.1%). Seven patients (17.9%) had mixed anaerobic bacteria. Two patients (5.2%) had a reported resistance to Clindamycin.

**Table 4.** Bacterial Culture and Antibiotic Resistance Results Based on Throat Swab or Peritonsillar Abscess Aspiration.

Variable	N (%) n = 39
Bacteria isolated	
No growth	14 (35.9)
*Streptococcus pyogenes*	12 (30.1)
*Streptococcus anginosus*	7 (17.9)
Mixed anaerobic bacteria	7 (17.9)
Group C Streptococcus	3 (7.7)
Normal oropharyngeal flora	2 (5.1)
Antibiotic resistance identified	
Clindamycin & penicillin	1 (2.6)
Clindamycin & erythromycin & tetracycline	1 (2.6)
Tetracycline	1 (2.6)

## Discussion

This is the first study examining the seasonal variation and epidemiology of PTA presentations to an ED in a maritime climate. The incidence of cases was highest in the summer and during warmer temperatures. Despite this, we found no significant seasonal or climate variation regarding the incidence of PTA.

PTA is theorized to be a progression of acute URTI.^1^ It has been hypothesized that the incidence of PTA would increase during colder months and the winter season when the incidence of URTI increases. However, our study and various others have found no significant seasonal variability.^2,9-13^ The few studies that did find significant seasonality, showed varying trends, with some noticing the inverse - increased incidence of PTA during the warmer summer season.^14-16^ Interestingly, though not significant, our patient cohort had a higher incidence of PTA in the summer.

Furthermore, some studies have shown an increased incidence of tonsillitis which did not correlate with increased cases of PTA.^1,15^ Based on this, it may be that acute URTI accounts for only a proportion of the pathogenesis of PTA cases. It has been suggested that blockage and damage to the minor salivary glands in the upper soft palate near the palatine tonsils, known as Weber’s glands, may be implicated in the pathogenesis of PTA.^1,8,21^ Klug et al suggested a unifying theory where acute URTI introduces the bacteria to the tonsils, and it spreads through the salivary system to the peritonsillar space.^22^ Although this study does not allow for further clarification of the pathogenesis of PTA, the lack of seasonal variation brings into question the classic thinking of solely progression from acute URTI.

Interestingly, most patients presenting in our study with a PTA were afebrile at the time of ED presentation. However, most patients did have an elevated WBC count. These characteristics are consistent with existing literature and suggest fever is not a reliable screening tool for PTA. However, the WBC count may support the diagnosis of PTA but not exclude it.^2^ The incidence of PTA peaked in those in their third decade of life (19-29), there was a slight male predominance, and there was a nearly equal proportion of PTA on either side of the oropharynx. These findings were consistent with previous epidemiological studies regarding PTA.^2,8-10^

In our cohort, 16.0% of patients were active smokers. This is only slightly increased compared to the 13.7% population prevalence of smoking in New Brunswick from 2017 data.^23^ Contrarily, Kilty and Gaboury^24^ reported smoking as a significant risk factor for PTA, with a 44% smoking rate in their cohort of patients with PTA from Ottawa, Ontario. However, a more recent study by Sowerby et al found a similar smoking rate to our study, at 15% of patients with PTA, which was lower than the prevalence of smoking in their study region, London, Ontario.^2^ It is possible that this represents the emphasis on reduction in smoking and increased smoking cessation in Canada or, due to the retrospective nature of our study, that the smoking rate was under-reported.

A major challenge in New Brunswick is timely access to primary care.^25^ We had hypothesized that those with more constrained access to primary care might have difficulty receiving treatment for oral cavity and oropharyngeal infections, thus resulting in an increased risk of PTA. However, almost all of the patients in our cohort were registered with a primary care provider.

Consistent with previous literature, Streptococcus species were the most common bacteria isolated.^2^ In our cohort, 17.9% of patients cultured had anaerobic bacteria present. This percentage is lower than the most recent Canadian literature, centered in London, Ontario, finding 44% anaerobic involvement.^2^ Our cohort is limited by the number of patients who had a throat swab or culture completed (n = 39, 59.0%). However, bacteriological studies are not found to impact decisions regarding the treatment of PTA and therefore may be unnecessary acute investigations and could be reserved for recurrent or challenging to treat abscesses.^26^

Clindamycin was the most prescribed antibiotic in our cohort in terms of antimicrobials. Interestingly, we did find that 5.2% (2/39) of isolated cultures were resistant to Clindamycin, while none of the cultures were resistant to Amoxicillin-Clavulanate. This resistance pattern is in keeping with recent reports of rising antibiotic resistance to Clindamycin and suggests we should consider a shift in our prescribing practice.^2,27,28^ Most of our patients were also given dexamethasone in the ED. Literature has shown that corticosteroids in PTA treatment reduce pain and improve clinical course.^29-31^

Like all research, our study is not without limitations. As our study was retrospective, certain descriptive variables were not routinely included in the ED charts and were not available for every patient. Specifically, data for bacteria isolated and antibiotic resistance was only available for 52.0% (n = 39) of patients and depended on the investigations ordered by the treating physician. We excluded patients <19 years old to limit confounding variables unique to children including exposure to crowded environments and their inherently immature immune systems. Furthermore, this study would not have captured patients with PTA that presented to primary care providers in the region outside of the. However, this study provides valuable information regarding the seasonal variation of PTA in a unique maritime climate, and it allows further insight into the limited literature surrounding the epidemiology of PTA in Canada.

## Conclusion

This study found no significant seasonal or climate variability of PTA at the SJRH ED. The lack of seasonality found in this, and other studies, brings into question the hypothesis of PTA pathogenesis from acute URTI. Future literature investigating the pathogenesis of PTA may provide valuable insight.
